# Microbial-Catalyzed Biotransformation of Multifunctional Triterpenoids Derived from Phytonutrients

**DOI:** 10.3390/ijms150712027

**Published:** 2014-07-07

**Authors:** Syed Adnan Ali Shah, Huey Ling Tan, Sadia Sultan, Muhammad Afifi Bin Mohd Faridz, Mohamad Azlan Bin Mohd Shah, Sharifah Nurfazilah, Munawar Hussain

**Affiliations:** 1 Faculty of Pharmacy, Universiti Teknologi MARA (UiTM), Puncak Alam Campus, 42300 Bandar Puncak Alam, Selangor Darul Ehsan, Malaysia; E-Mails: fifa200208@gmail.com (M.A.B.M.F.); benzene301@gmail.com (M.A.B.M.S.); sharifahnurfazilah@hotmail.com (S.N.); 2 Atta-ur-Rahman Institute for Natural Products Discovery (AuRIns), Level 9, FF3, Universiti Teknologi MARA (UiTM), Puncak Alam Campus, 42300 Bandar Puncak Alam, Selangor Darul Ehsan, Malaysia; 3 Faculty of Chemical Engineering, Universiti Teknologi MARA (UiTM), 40450 Shah Alam, Selangor Darul Ehsan, Malaysia; 4 Department of Basic Sciences, DHA Suffa University, Off, Khayaban-e-Tufail, Phase VII (Extension), DHA, Karachi 75500, Pakistan; E-Mail: mhhej@yahoo.co.in

**Keywords:** microbial transformation, tetranortriterpenoids, tetracyclic triterpenoids, pentacyclic triterpenoids, biocatalysis

## Abstract

Microbial-catalyzed biotransformations have considerable potential for the generation of an enormous variety of structurally diversified organic compounds, especially natural products with complex structures like triterpenoids. They offer efficient and economical ways to produce semi-synthetic analogues and novel lead molecules. Microorganisms such as bacteria and fungi could catalyze chemo-, regio- and stereospecific hydroxylations of diverse triterpenoid substrates that are extremely difficult to produce by chemical routes. During recent years, considerable research has been performed on the microbial transformation of bioactive triterpenoids, in order to obtain biologically active molecules with diverse structures features. This article reviews the microbial modifications of tetranortriterpenoids, tetracyclic triterpenoids and pentacyclic triterpenoids.

## 1. Introduction

Natural products extracted from plants, marine sources and microorganisms constitute a rich source of diverse scaffolds for drug discovery. Often they form the backbone of innovative drug discovery programs. They can either be directly used as drugs to treat various diseases, or used as a valuable starting material (“lead”) for drug discovery process. From the 1940s until 2010, 65% of antibacterial and 41% of anticancer small molecule drugs developed were either natural products or semi-synthetic derivatives of natural products [1,2,3,4]. Structural diversification of multifunctional natural products is often required to improve their solubility, reduce toxicity, or enhance efficacy. Chemical conversions may provide abundant products, but are limited by regio- and stereoselectivity constraints. Moreover, multi-step chemical reactions often result in low overall yield of the final products. However, biocatalytic reactions are well-established “green” techniques for carrying out high chemo-, regio- and stereoselective functionalization of various sensitive and complex molecules under mild reaction conditions and hence are much more attractive for drug development process [5,6,7,8]. Biocatalysis, using multi-enzyme systems of fungi, bacteria, and cultured plant suspension cells has the advantage of producing compounds with high selectivity and efficiency under mild conditions. Therefore, biological systems are widely used in the pharmaceutical industry [9,10,11,12,13,14,15,16,17,18,19,20,21]. The use of microorganisms such as bacteria and fungi as a biocatalytic system imitates the mamalian metabolism to perform selective transformation reactions and improve the economically and ecologically friendly microbial transformations [22,23,24]. A number of filamentous fungi are known to perform complex biotransformations that are difficult to achieve by chemical means [22,23,24,25,26]. Many microorganisms, especially certain filamentous fungi, have the ability to transform terpenoids chemo-, regio- and stereoselectively. The fungal-mediated oxidation of terpene under mild conditions appears as an attractive alternative as compared to the traditional chemical methods, have an elevated chemo-, regio- and enantioselectivity, and do not generate toxic waste products, and the products obtained can be labeled as “natural” source [5]. Microbial cell-mediated transformations have been extensively used for *in vitro in vitro* drug metabolic studies. Moreover, microbial transformations can also provide better yields of the metabolites with high selectivity for toxicological and biological studies [22]. Fungi also provide additional advantage in performing reactions similar to mammalian transformations [24].

Triterpenes are plant-derived natural compounds built-up from six isoprene units (C_5_H_8_), while triterpenoids consist of both the basic triterpene skeleton and their derivatives that contain oxygen moiety. The simplest triterpene with skeletal structure that forms basis for complex triterpenoids is squalene (C_30_) [8]. It forms the precursor for a structurally diverse group of natural products that display nearly 200 distinct skeletons. These products have been studied for their antiviral (anti-HIV), antineoplastic, anti-inflammatory, anti-ulcerogenic, antimicrobial, anti-plasmodial, hepato- and cardio-protective, analgesic, anti-mycotic, and immunomodulatory effects [1,2,6,7,8]. They are routinely found in numerous medicinal plants. They are also excellent starting material for the synthesis of many fine chemicals due to their homogeneous carbon skeleton [1]. Microbial cell-based transformations of triterpenoids have been developed primarily in the past two decades to produce novel lead molecules, new pharmaceuticals, and agrochemical compounds [7,8,27,28,29,30,31]. Some synthetic oleanane triterpenoids derived from microbial transformation act as multifunctional drugs that regulate the activity of entire networks [32,33,34,35,36]. Microbial biocatalysis has already been proven as powerful tool in the generation of structural diversity in triterpenoid skeletons for future structure-activity relationship studies [5,6,7,8].

**Figure 1:**
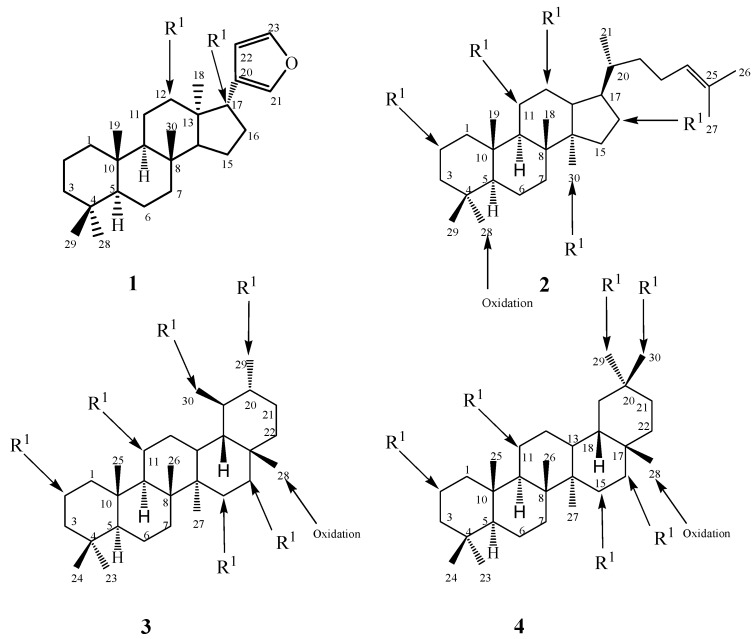
Structures of tetranortriterpenoids (**1**), tetracyclic triterpenoids (**2**), pentacyclic triterpenoids (**3**–**4**) and microbial target positions of substituents.

This review provides an overview of the structures of diverse and novel products obtained during biotransformation of multifunctional triterpenoid drugs with growing cultures of fungi and bacteria. Different microbial cultures and reaction conditions used in biotransformation of triterpenoid drugs and structure determination methods used in biotransformational processes are discussed.

## 2. Fungal Culture Regioselectivity on Triterpenoid Skeleton

Fungi have been described as useful tool for the biotransformation of natural and semisynthetic triterpenoids [7,8]. Nevertheless, in most of the examples, only minor or simple transformations of functional groups have been detected [6,9].

**Figure 2:**
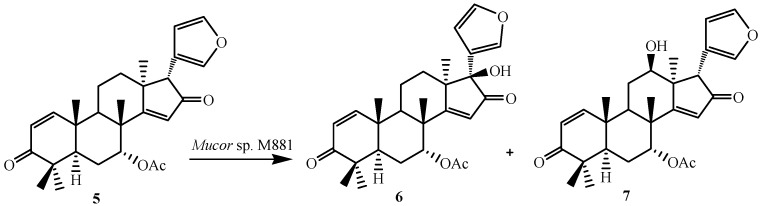
Biotransformation of azadiradione (**5**) by *Muco*r sp. M881.

**Figure 3:**
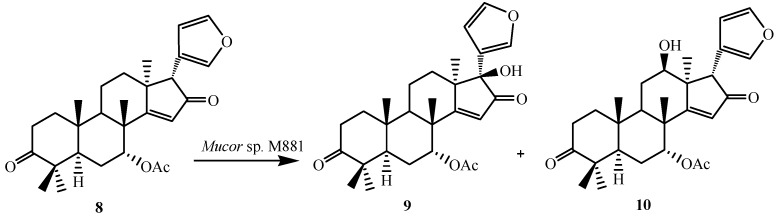
Biotransformation of 1,2-dihydroazadiradione (**8**) by *Muco*r sp. M881.

**Figure 4:**
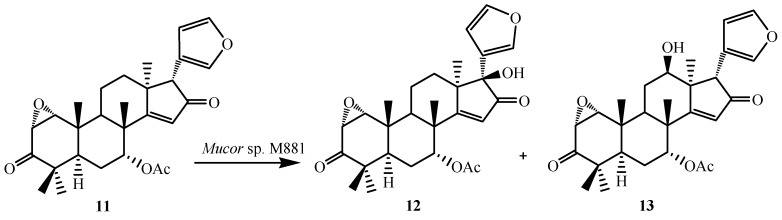
Biotransformation of 1,2α-epoxyazadiradione (**11**) by *Muco*r sp. M881.

**Figure 5:**
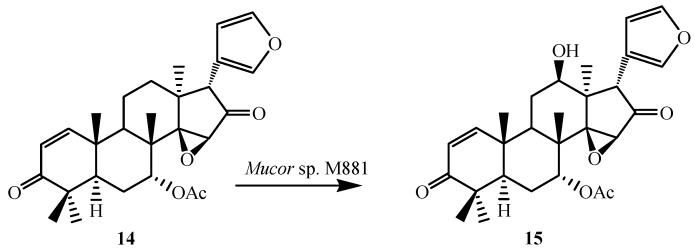
Biotransformation of 14,15β-epoxyazadiradione (**14**) by *Muco*r sp. M881.

**Figure 6:**
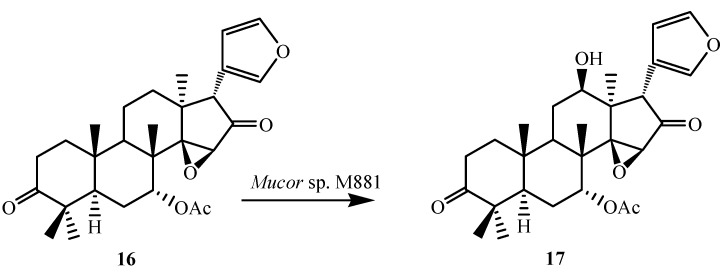
Biotransformation of 1,2-dihydroepoxyazadiradione (**16**) by *Muco*r sp. M881.

**Figure 7:**
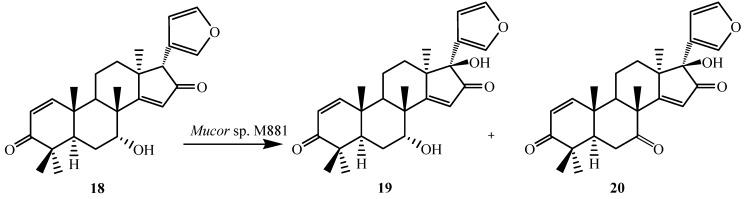
Biotransformation of 7-deacetylazadiradione (**18**) by *Muco*r sp. M881.

**Figure 8:**
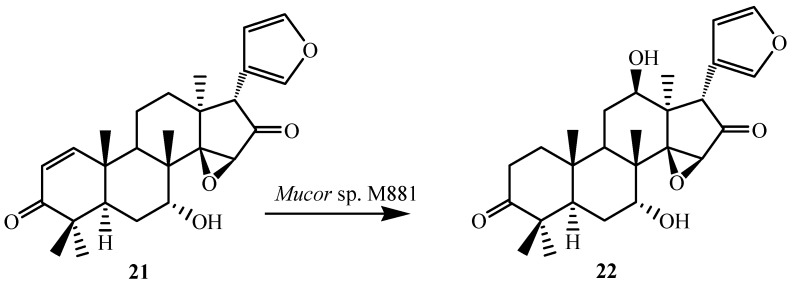
Biotransformation of 7-deacetylepoxyazadiradione (**21**) by *Muco*r sp. M881.

**Figure 9:**
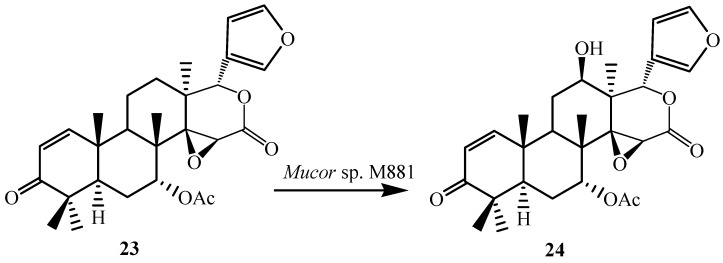
Biotransformation of gedunin (**23**) by *Muco*r sp. M881.

Microbial cell cultures are capable of performing specific chemical transformations in triterpenoids, such as rearrangement, hydroxylation, oxidation, reduction, hydrolysis, epimerization and isomerization, with high regio- and stereoselectivity as shown in Figure 1 [6,7,8]. In Figure 2, Figure 3, Figure 4, Figure 5, Figure 6, Figure 7, Figure 8 and Figure 9, we can see a variation in biocatalytic system introduce regio-selectivity among 12β- or 17β-hydroxylation in limonoids skeletons [23]. *Cunninghamella elegans* AS 3.1207 also transforms steroidal saponins into pregnenolones, *S. racemosum* AS 3.264 converts paeoniflorin into albiflorin [26]. *Cunninghamella blakesleeana* NRRL 1369 performs complicated rearrangement of tetracyclic triterpenoids into novel ranunculane framework, which reveals the biocatalytic potential of microorganisms in diversification and promoting structural transformation [22,23,24,25,26].

## 3. Microbial-Catalyzed Biotransformation of Tetranortriterpenoids

Limonoids (**1**), chemically classified as tetranortriterpenoids, are metabolically modified triterpenes having an intact 4,4,8-trimethyl-17-furanylsteroid precursor skeleton (basic limonoid). In some cases, the skeleton is further rearranged and highly oxygenated, creating a structural diversity. They are known to possess anti-cancer, anti-malarial, anti-HIV, antimicrobial and several other pharmacological activities. Azadiradione (**5**), epoxyazadiradione (**14**), gedunin (**23**) and their derivatives fall under this group. The strong antifeedant properties along with anti-plasmoidal, anti-HIV, and anti-inflammatory activities of azadiradione and epoxyazadiradione have attracted the attention of synthetic chemists in the last two decades [23]. In particular, gedunin (**23**) is a well-studied anti-malarial, anti-carcinogenic and antiulcerogenic agent.

Thulasiram *et al.* developed a highly efficient fungi-mediated bioconversion for the 12β- and 17β-hydroxylation of the basic limonoid family of compounds (Figure 2). The fungal system belonging to the genera of *Mucor* (National Collection of Industrial Microorganisms or NCIM, Pune, catalogue no. 881 and abbreviated as M881) efficiently transformed azadiradione (**5**), epoxyazadiradione (**14**), gedunin (**23**) and their derivatives (**8**, **11**, **16**, **18** and **21**) into corresponding 12β- and/or 17β-hydroxy derivatives. These microbial-catalyzed stereo- and regioselective hydroxylation of limonoid skeleton was highly efficient in introducing chemically sensitive functional moieties [23].

Azadiradione (**5**), a limonoid isolated from the fruit of *Azadirachta indica* (Neem). The fungus *Mucor* sp. M881 regioselectively transformed Azadiradione (**5**), an isolated limonoid from the fruit of *Azadirachta indica* (Neem), to 17β-hydroxyazadiradione (**6**) and 12β-hydroxyazadiradione (**7**) (Figure 2).

To further investigate the substrate specificity of the organism, the biotransformation was studied with seven natural or semi-synthetic derivatives of azadiradione (**8**, **11**, **14**, **16**, **18**, **21** and **23**) (see Figure 3, Figure 4, Figure 5, Figure 6, Figure 7, Figure 8 and Figure 9). Fungi-mediated biocatalysis of 1,2-dihydroazadiradione (**8**) and 1,2α-epoxyazadiradione (**11**) resulted in regioselective hydroxylation at C-17β- and C-12β-sites (**9**, **10**, **12** and **13**) with excellent yields. These biotransformation reactions are shown in Figure 3 and Figure 4. Fermentation of 14,15β-epoxyazadiradione (**14**) and 1,2-dihydroepoxyazadiradione (**16**) with *Mucor* sp. M881 (see Figure 5 and Figure 6) afforded regioselective hydroxylation specifically at the C-12β site (**15** and **17**). The organism also hydroxylated gedunin (**23**, expanded D ring as six membered lactone) and produced 12β-hydroxy gedunin (**24**) as the sole biotransformed product (Figure 9) [23].

Similarly, the *Mucor* sp. M881 was able to hydroxylate 7-deacetylepoxyazadiradione (**21**) at the 12β-position (**22**) (Figure 8). Nevertheless, when 7-deacetylazadiradione (**18**, nimbocinol) was used as a substrate (Figure 7), this compound was hydroxylated at the 17β-position to produce 17β-hydroxynimbocinol (**19**) along with another product 7-oxo-17β-hydroxynimbocinol (**20**) as metabolites. These results seems to indicate that the two functional groups (epoxy group in ring D and acetate in ring B) are critical in producing different products (17β-hydroxy:12β-hydroxy). All these metabolites (**6**–**24**) were characterised by detailed ^1^H and ^13^C NMR spectroscopy. The location of the hydroxyl functionalities were deduced on the basis of the heteronuclear multiple bond connectivity (HMBC) interactions whereas orientation of OH groups was deduced on the basis of Nuclear Overhauser Effect spectroscopy (NOESY) correlations [23].

## 4. Microbial-Catalyzed Biotransformation of Natural Tetracyclic Triterpenoids

**Figure 10:**
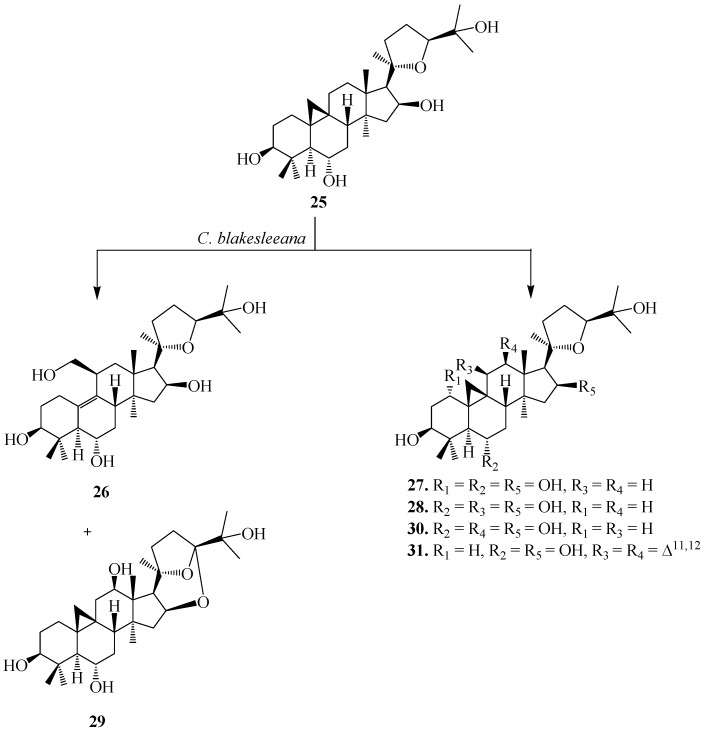
Biotransformation of cycloastragenol (**25**) by *Cunninghamella blakesleeana* NRRL 1369.

Cycloastragenol (**25**), or (20*R*,24*S*)-3β,6α,16β,25-tetrahydroxy-20,24-epoxycycloartane (Figure 10), is the genuine sapogenin of astragaloside IV, a major bioactive constituent of Astragalus plants. Astragaloside IV exhibits various pharmacological properties, such as anti-inflammatory, anti-viral, anti-aging and anti-oxidant. It could retard the onset of cellular aging by progressing telomerase activity, up-regulate the immune system by inducing IL-2 release, and elevate the antiviral function of human CD8+ T lymphocytes. Compound **25** has been considered as a promising new generation of anti-aging agent [26].

**Figure 11:**
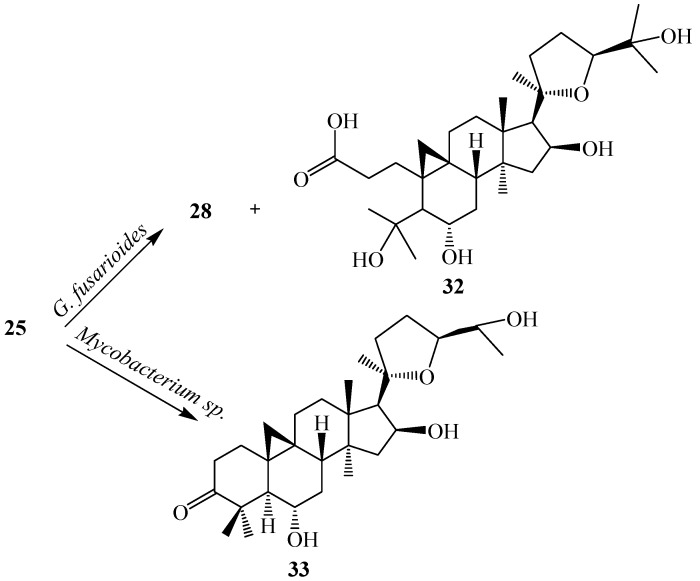
Biocatalytic reactions of *Glomerella fusarioides* ATCC 9552 and *Mycobacterium* sp. NRRL 3805 on cycloastragenol (**25**).

The biocatalysis of **25** with two fungal strains, *Cunninghamella blakesleeana* NRRL 1369 and *Glomerella fusarioides* ATCC 9552, and the bacterium *Mycobacterium* sp. NRRL 3805 was investigated by Kuban *et al.* [24,25]. These strains efficiently transformed **25** into hydroxylated metabolites along with products formed by cyclization, dehydrogenation and Baeyer–Villiger oxidation resulting in a ring cleavage (Figure 10) [25]. *C. blakesleeana* efficiently transformed **25** into a complicated rearrangement product, *i.e.*, ring cleavage and methyl group migration of **25**, (20*R*,24*S*)-3β,6α,6β,19,25-pentahydroxy-ranunculan-9(10)-ene (**26**) (Figure 10) [24]. With same microorganism, the substrate cycloastragenol (**25**) underwent several regioselective hydroxylatedproducts, (20*R*,24*S*)-3β,6α,16β,19,25-pentahydroxy-ranunculan-9(10)-ene (**26**), (20*R*,24*S*)-epoxy-1α-3β,6α,16β,25-pentahydroxycycloartane (**27**), (20*R*,24*S*)-epoxy-3β,6α,11β,16β,25-pentahydroxycycloartane (**28**), (20*R*,24*S*)-16β,24;20,24-diepoxy-3β,6α,12β,25-tetrahydroxycycloartane (**29**), (20*R*,24*S*)-epoxy-3β,6α,12β-16β,25-pentahydroxycycloartane (**30**) and an unsaturated product, (20*R*,24*S*)-epoxy-3β,6α,16β,25-tetrahydroxycycloarta-11(12)-ene (**31**). Ring cleavage product, 3,4-seco cycloastragenol (**32**) and (20*R*,24*S*)-epoxy-3β,6α,11β,16β,25-pentahydroxycycloartane (**28**) was isolated from the fungus *G. fusarioides*.The *Mycobacterium* sp. NRRL 3805 transformed **25** into oxidation product, **33** in 24 h. These biotransformation reactions are shown in Figure 10 and Figure 11 [25].

**Figure 12:**
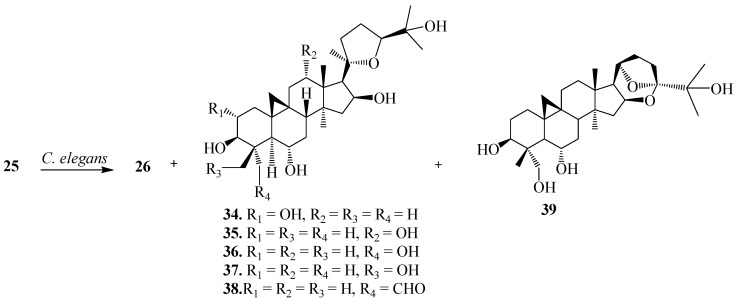
Biocatalytic reactions of *Cunninghamella elegan*s AS 3.1207 on cycloastragenol (**25**).

Ye *et al.* reported the biotransformation of **25** by *Cunninghamella elegans* AS 3.1207, *Syncephalastrum racemosum* AS 3.264 and *Doratomyces stemonitis* AS 3.1411 [26]. Biocatalytic fermentations of **25** with *C. elegans* AS 3.1207 for 6 days afforded several regioselective hydroxylated metabolites presented in Figure 12, (20*R*,24*S*)-2α,3β,6α,16β,25-pentahydroxy-20,24-epoxycycloartane (**34**), (20*R*,24*S*)-3β,6α,12α,16β,25-pentahydroxy-20,24-epoxy-cycloartane (**35**), (20*R*,24*S*)-3β,6α,16β,25,28-pentahydroxy-20,24-epoxy-cycloartane (**36**), (20*R*,24*S*)-3β,6α,16β,25,29-pentahydroxy-20, 24-epoxy-cycloartane (**37**), (20*R*,24*S*)-3β,6α,16β,25-tetrahydroxy-20,24-epoxy-cycloartan-28-carbaldehyde (**38**), (20*R*,24*R*)-3β,6α,25,28-tetrahydroxy-16β,24:20,24-diepoxy-cycloartane (**39**) and (20*R*,24*S*)-3β,6α,16β,19,25-pentahydroxy-ranunculan-9(10)-ene (**26**) [26].

**Figure 13:**
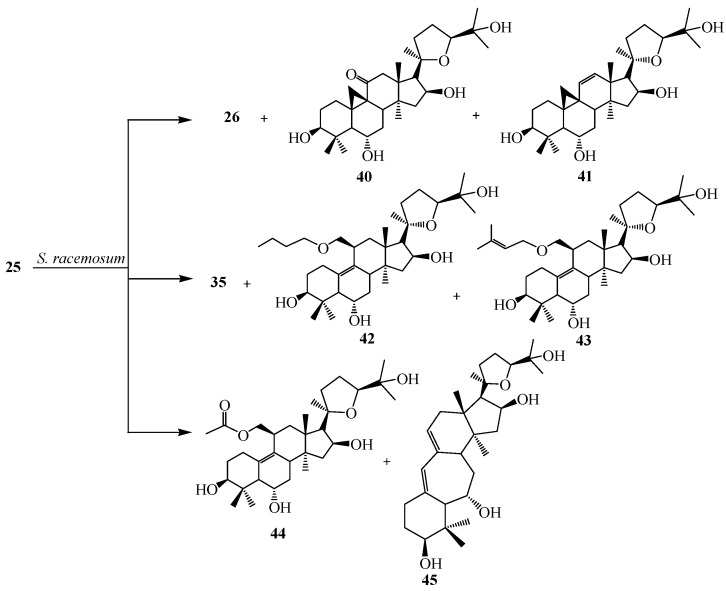
Biotransformation of *Syncephalastrum racemosum* AS 3.264 on cycloastragenol (**25**).

**Figure 14:**
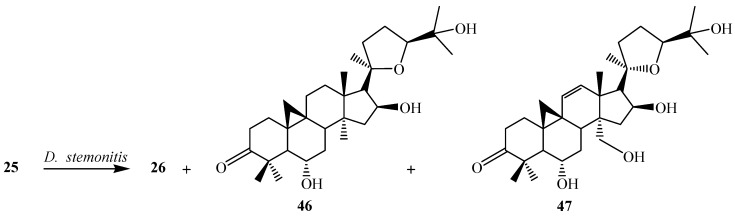
Biotransformation of *Doratomyces stemonitis* AS 3.1411 on cycloastragenol (**25**).

Bioconversion of **25** by *S. racemosum* AS 3.264 yielded (20*R*,24*S*)-3β,6α,16β,19,25-pentahydroxy-ranunculan-9(10)-ene (**26**), (20*R*,24*S*)-3β,6α,12α,16β,25-pentahydroxy-20,24-epoxy-cycloartane (**35**), (20*R*,24*S*)-3β,6α,16β,25-tetrahydroxy-20,24-epoxy-cycloartan-11-one (**40**), (20*R*,24*S*)-3β,6α,16β,25-tetrahydroxy-20,24-epoxy-cycloartan-11(12)-ene (**41**), (20*R*,24*S*)-3β,6α,16β,25-tetrahydroxy-19-butoxy-ranunculan-9(10)-ene (**42**), (20*R*,24*S*)-3β,6α,16β,25-tetrahydroxy-19-isopentenyloxyranunculan-9(10)-ene (**43**), (20*R*,24*S*)-3β,6α,16β,25-tetrahydroxy-19-acetoxy-ranunculan-9(10)-ene (**44**) and ring expansion metabolite, neoastragenol or (20*R*,24*S*)-3β,6α,16β,25-tetrahydroxy-20,24-epoxy-9(10)a-homo-19-nor-cycloartane (**45**) (Figure 13). *D. stemonitis* AS 3.1411 transformed **25** to two carbonylated metabolites, (20*R*,24*S*)-6α,16β,25-trihydroxy-20,24-epoxy-cycloartan-3-one (**46**), (20*R*,24*S*)-6α,16β,25,30-tetrahydroxy-20,24-epoxy-cycloartan-3-one (**47**) and **26**. These transformations are shown in Figure 14 [26].

**Figure 15:**
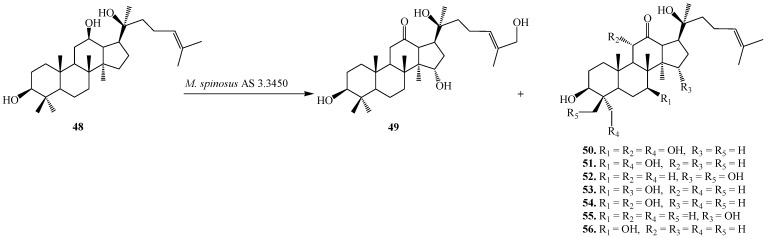
Biotransformation of *Mucor spinosus* AS 3.3450 on 20(*S*)-protopanaxadiol (**48**).

**Figure 16:**
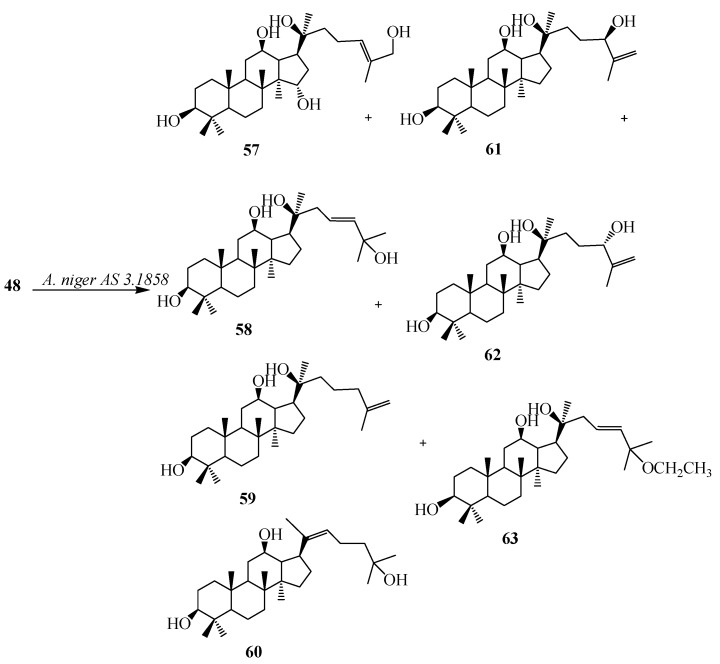
Biotransformations of *Aspergillus niger* AS 3.1858 on 20(*S*)-protopanaxadiol (**48**).

The tetratriterpenoid 20(*S*)-Protopanaxadiol (**48**), a glycone of ginsenosides from the Chinese ginseng (*Panax ginseng* C.A. Mey, Araliaceae) exhibits powerful pleiotropic anti-cancer effects in several cancer cell lines including the inhibition of metastasis. Furthermore, compound **48** could induce apoptosis through mitochondria mediated apoptotic pathway in Caco-2, U937, THP-1, and SMMC7721 cancer cells [27]. The fungal tansformation of **48** was reported by Li *et al.* Fermentation of **48** with *Mucor spinosus* AS 3.3450 for 5 days yielded eight regioselective hydroxylated metabolites (**49**–**56**), 12-oxo-15α,27-dihydroxyl-20(*S*)-protopanaxadiol (**49**), 12-oxo-7β,11α,28-trihydroxyl-20(*S*)-protopanaxadiol (**50**), 12-oxo-7β,28-dihydroxyl-20(*S*)-protopanaxadiol (**51**), 12-oxo-15α, 29-dihydroxyl-20(*S*)-protopanaxadiol (**52**), 12-oxo-7β,15α-dihydroxyl-20(*S*)-protopanaxadiol (**53**), 12-oxo-7β,11β-dihydroxyl-20(*S*)-protopanaxadiol (**54**), 12-oxo-15α-hydroxyl-20(*S*)-protopanaxadiol (**55**), and 12-oxo-7β-hydroxyl-20(*S*)-protopanaxadiol (**56**) (Figure 15). Incubation of **48** with *Aspergillus niger* AS 3.1858 afforded seven additional hydroxylated metabolites (**57–63**), 26-hydroxyl-20(*S*)-protopanaxadiol (**57**), 23,24-en-25-hydroxyl-20(*S*)-protopanaxadiol (**58**), 25,26-en-20(*S*)-protopanaxadiol (**59**), (*E*)-20,22-en-25-hydroxyl-20(*S*)- protopanaxadiol (**60**), 25,26-en-24(*R*)-hydroxyl-20(*S*)-protopanaxadiol (**61**), 25,26-en-24(*S*)-hydroxyl-20(*S*)-protopanaxadiol (**62**) and 23,24-en-25-ethoxyl-20(*S*)-protopanaxadiol (**63**) [28]. These biotransformation reactions are described in Figure 16.

**Figure 17:**
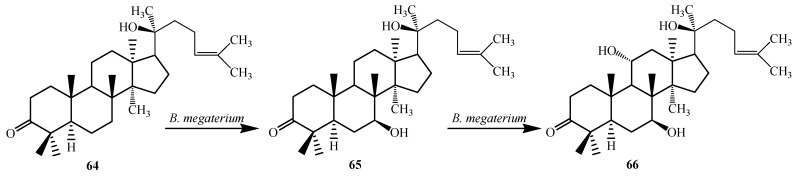
Biotransformation of dipterocarpol (**64**) with *Bacillus megaterium* ATCC 13368.

The bacterium *Bacillus megaterium* metabolized the triterpenoid dipterocarpol (**64**) to 7β-hydroxydipterocarpol (**65**) and 7β,11α-dihydroxydipterocarpol (**66**) in 16 h (Figure 17). The Dipterocarpol (**64**) and the dihydroxylated product **66** did not displayed cytotoxic activity with HeLa and COS-1 cells while 7β-hydroxylated product **65** exhibited cytotoxicity on both the cell lines [29].

**Figure 18:**
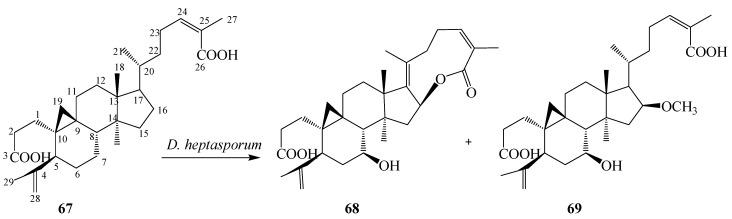
Microbiological transformation of the triterpene nigranoic acid (**67**) by the freshwater fungus *Dictyosporium heptasporum*.

*Schisandra propinqua* var. *sinensis*, popularly known as “tie-gu-san” produced by Schisandraceae family in the Shennongjia district of mainland China, is used as folk medicine for the treatment of arthritis, traumatic injury, gastralgia, angeitis, and other related diseases. Nigranoic acid (3,4-secocycloarta- 4(28),24(*Z*)-diene-3,26-dioic acid, **67**) is the first member of class 3,4-secocycloartane triterpenoid produced by *Schisandra propinqua*, that has been reported to possess a variety of biological activities, including cytotoxic activity toward leukemia and Hela cells, and inhibition of expression of HIV reverse transcriptase and polymerase [31]. Dong *et al.* studied the microbiological transformation of **67** by the freshwater fungus *Dictyosporium heptasporum* YMF1.01213. The organism metabolized **67** into novel nine-membered lactone ring 3,4-secocycloartane triterpenoid derivatives, 3,4-secocycloarta-4(28),17(20),24(*Z*)-triene-7β-hydroxy-16β,26-lactone-3-oic acid (**68**) and 3,4-secocycloarta-4(28),17(20)(*Z*),24(*Z*)-triene-7β-hydroxy-16β-methoxy-3,26-dioic acid (**69**) (Figure 18) [30].

**Figure 19:**
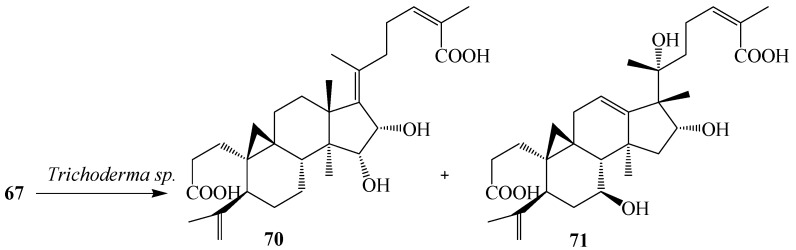
Metabolism of **67** by *Trichoderma* sp. JY-1 culture.

Dong *et al.* reported the bioconversion of **67** by cultures of *Trichoderma* sp. JY-1. The fungus yielded 15α,16α-dihydroxy-3,4-secocyloarta-4(28),17(20),17(*E*),24(*E*)-triene-3,26-dioic acid (**70**) and 16α,20α-dihydroxy-18 (13→17β) abeo-3,4-secocyloarta-4 (28),12(13),24(*Z*)-triene-3,26-dioic acid (**71**). Substrate **67** and its transformed products **70** and **71** displayed weak anti-HIV activity with EC_50_ values of 10.5, 8.8 and 7.6 mg/mL, and therapeutic index values (CC_50_/EC_50_) of 8.48, 9.12 and 10.1, respectively (Figure 19) [31].

## 5. Microbial Transformation of Natural and Semi-Synthetic Pentacyclic Triterpenoids

Pentacyclic triterpenoids (**3**–**4**) are widely distributed in many medicinal plants, such as birch bark (betulin, **153**), plane bark (betulinic acid, **154**), olive leaves, olive pomace, mistletoe sprouts and clove flowers (oleanolic acid, **72**), and apple pomace (ursolic acids, **114**). Compounds belonging to this group such as lupane (lupeol, betulin, betulinic acid), oleanane (**72** and maslinic acid (**73**), erythrodiol, β-amyrin), and ursane (**114**, uvaol, α-amyrin) display various pharmacological effects. These triterpenoids are ideal and potential candidates for designing lead compounds for the development of new bioactive agents [32].

Olean-type pentacyclic triterpenes (OPTs) are plant-derived natural products, abundantly found in many medicinal herbs. They display a remarkable spectrum of biological activities, such as antimalarial, anti-tumor, anti-HIV, anti-microbial, anti-diabetic, and anti-inflammatory activities [32]. The microbial-catalyzed modification of olean-type pentacyclic triterpenes mainly resulted in the substitution of hydroxyl or carbonyl groups to the methyl or methenyl carbons of the skeleton and the formation of corresponding glycosides [32]. The presence of such functional moieties, especially at C-3, C-28, and C-30, seems to contribute to the biological activities of pentacyclic triterpenoids [17,35,36].

Oleanolic acid (3β-hydroxyolean-12-en-28-oic acid, **72**) is a natural pentacyclic triterpenoid compounds widely present in the form of free acid or aglycone of triterpenoid saponins. It is usually found in high concentrations in olive-pomace oil [32]. Some oleanolic acids have been reported to be antimalarial, antitumor, hepatoprotective, anti-HIV, and skin protective. They seem to possess α-glucosidase inhibitory activity.

**Figure 20:**
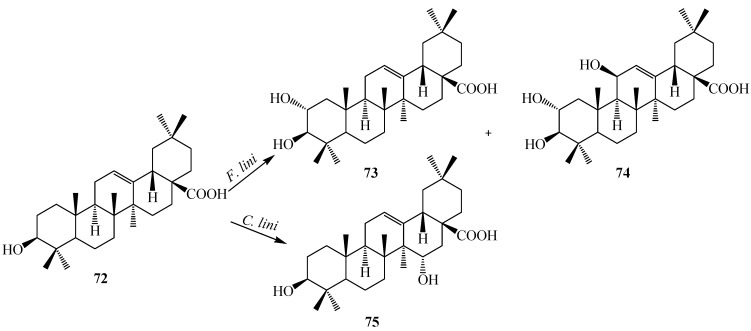
Microbial transformation of oleanolic acid (**72**) by *Furarium lini* NRRL-68751 and *Colletotrichum lini* AS3.4486.

Several biotransformations of oleanolic acid (**72**) with different microorganisms have been reported so far. Choudhary *et al.* investigated the metabolism of oleanolic acid (**72**) with *Fusarium lini* NRRL-68751 and reported the production of two oxidative metabolites, maslinic acid (2α,3β-dihydroxyolean-12-en-28-oic acid, **73**) and 2α,3β,11β-trihydroxyolean-12-en-28-oic acid (**74**) (Figure 20). These metabolites show potent inhibition of α-glucosidase activity. Hua *et al.* reported the metabolism of **72** with *Colletotrichum lini* AS3.4486 which resulted in one polar metabolite, 15α-hydroxyl-oleanolic acid (**75**) (Figure 20) [17,33].

**Figure 21:**
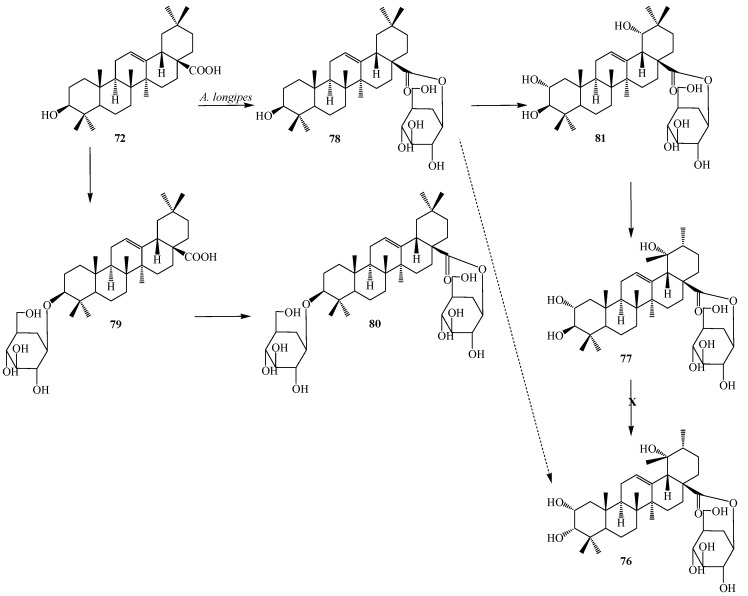
Microbial transformation of oleanolic acid (**72**) by *Alternaria longipes*.

**Figure 22:**
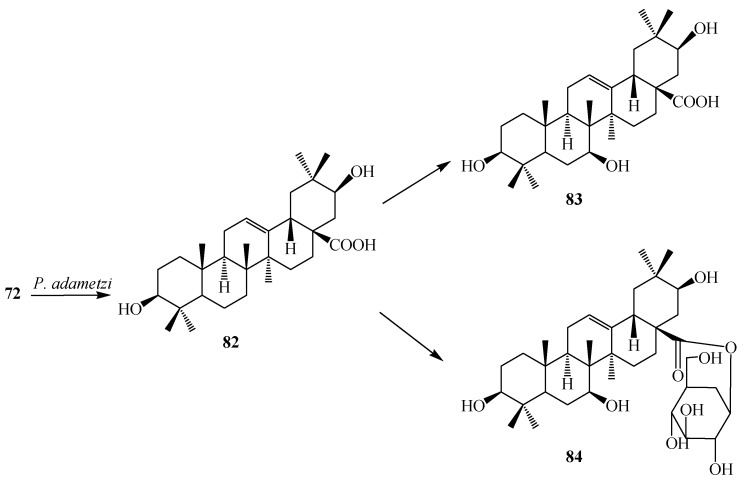
Microbial transformation of oleanolic acid (**72**) by *Penicillium adametzi*.

Liu *et al.* reported microbial transformation of **72** with *Alternaria longipes* and *Penicillium adametzi*. The fungus *Alternaria longipes* converted **72** to six different regioselective hydroxylated products, 2α,3α,19α-trihydroxy-ursolic acid-28-*O*-β-d-glucopyranoside (**76**), 2α,3β,19α-trihydroxy-ursolic acid-28-*O*-β-d-glucopyranoside (**77**), oleanolic acid 28-*O*-β-d-glucopyranosyl ester (**78**), oleanolic acid-3-*O*-β-d-glucopyranoside (**79**), 3-*O*-(β-d-glucopyranosyl)-oleanolic acid-28-*O*-β-d-glucopyranoside (**80**), 2α,3β,19α-trihydroxy-oleanolic acid-28-*O*-β-d-glucopyranoside (**81**), while cultures of *Penicillium adametzi* transformed the **72** to 21β-hydroxyl oleanolic acid (**82**), 7α,21β-dihydroxyl oleanolic acid (**83**) and 21β-hydroxyl oleanolic acid-28-*O*-β-d-glucopyranoside (**84**). These fermentation reactions are presented in Figure 21 and Figure 22. Compounds **79** and **82**–**84** exhibited stronger cytotoxic activities against Hela cell lines than the substrate **72** [34].

**Figure 23:**
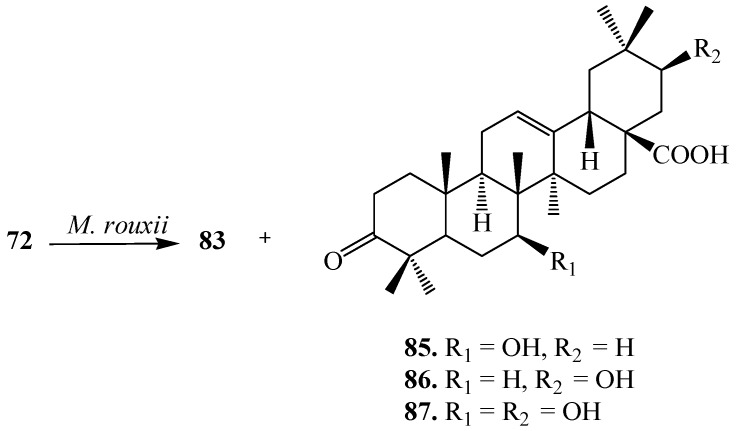
Microbial transformation of oleanolic acid (**72**) by *Mucor rouxii*.

Chapel *et al.* reported the microbial transformation of **72** with the filamentous fungus, *Mucor rouxii*. Fermentation process yielded four regioselective derivatives, **83**, 7*β*-hydroxy-3-oxo-olean-12-en-28-oic acid (**85**), 21*β*-hydroxy-3-oxo-olean-12-en-28-oic acid (**86**) and 7*β*,21*β*-dihydroxy-3-oxo-olean-12-en-28-oic acid (**87**) (Figure 23). Compound **86** displayed the potent activity against *Porphyromonas gingivalis* [35].

**Figure 24:**
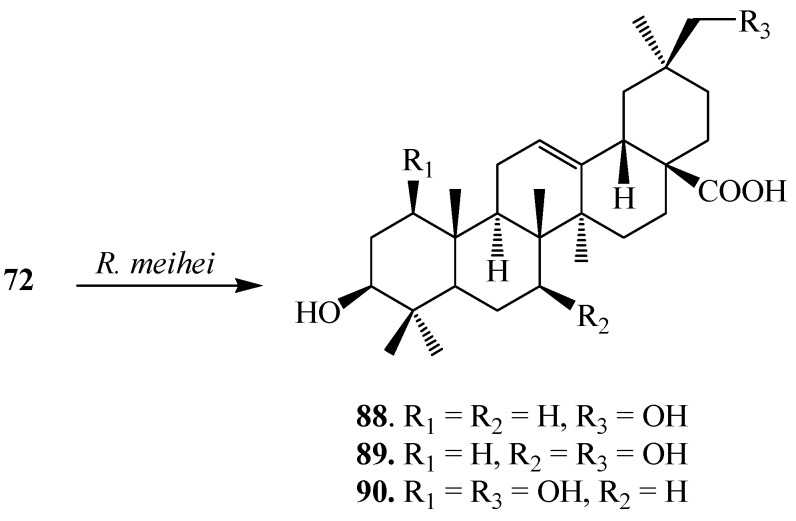
Metabolites isolated from the incubation of oleanolic acid (**72**) with *Rhizomucor miehei*.

**Figure 25:**
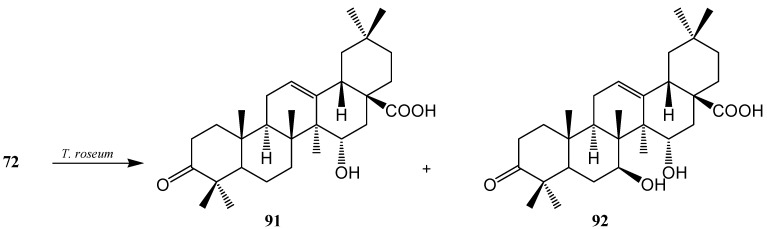
Metabolites isolated from the incubation of oleanolic acid (**72**) with *Trichothecium roseum*.

Martinez *et al.* investigated biotransformation of **72** with *Rhizomucor miehei* CECT 2749 and reported the production of a mixture of polar metabolites, 3β,30-dihydroxyolean-12-en-28-oic acid (**88**), (also called queretaroic acid), 3β,7β,30-trihydroxyolean-12-en-28-oic acid (**89**), (also called canthic acid) and 1β,3β,30-trihydroxyolean-12-en-28-oic acid (**90**) (Figure 24) [36]. On the other hand, Gong *et al.* reported metabolism of **72** with *Trichothecium roseum* and showed the production of two hydroxylated metabolites, 15α-hydroxy-3-oxo-olean-12-en-28-oic acid (**91**) and 7β,15α-dihydroxy-3-oxo-olean-12-en-28-oic acid (**92**) (Figure 25) [37].

**Figure 26:**
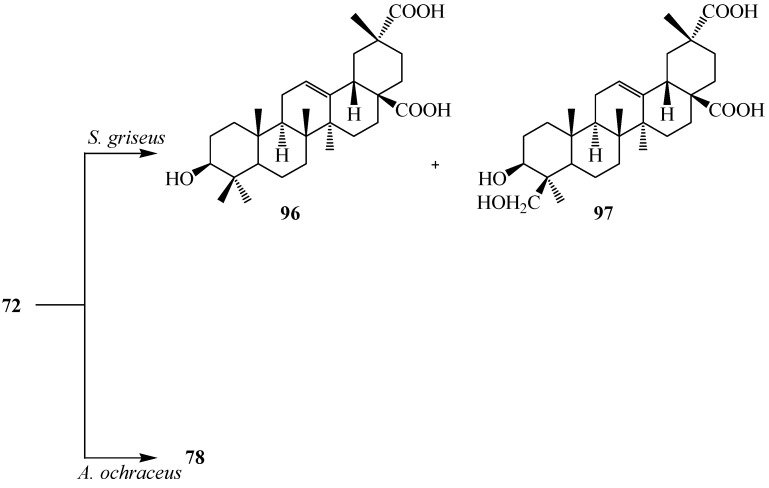
Microbial transformation of oleanolic acid (**72**) by *Streptomyces griseus* ATCC 13273 and *Aspergillus ochraceus* CICC 40330.

**Figure 27:**
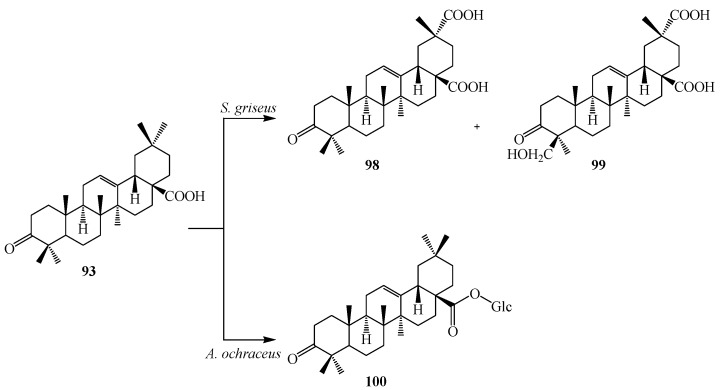
Microbial transformation of 3-oxo oleanolic acid (**93**) by *Streptomyces griseus* ATCC 13273 and *Aspergillus ochraceus* CICC 40330.

**Figure 28:**
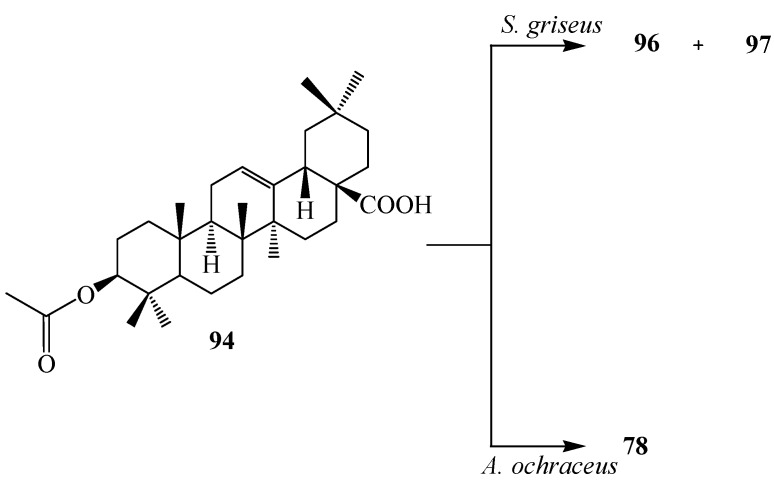
Microbial transformation of 3-acetyl oleanolic acid (**94**) by *Streptomyces griseus* ATCC 13273 and *Aspergillus ochraceus* CICC 40330.

**Figure 29:**
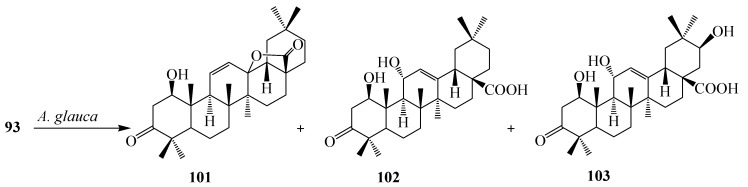
Microbial transformation of 3-oxo oleanolic acid (**93**) by growing cultures of the fungus *Absidia glauca*.

**Figure 30:**
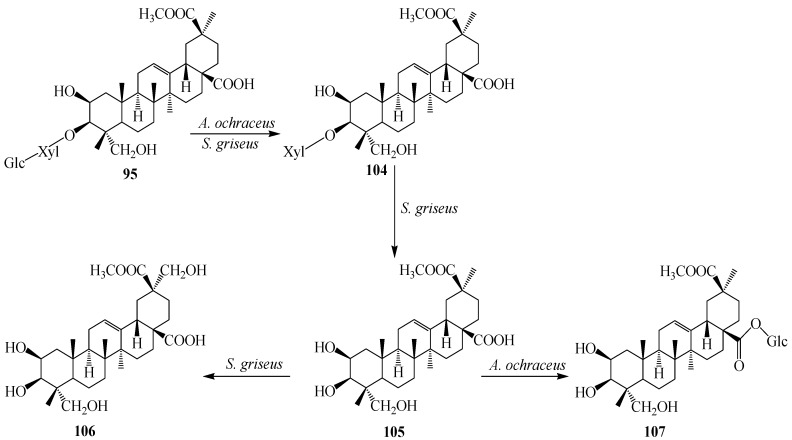
Microbial transformation of 3-oxo esculentoside A (**95**) by *Streptomyces griseus* ATCC 13273 and *Aspergillus ochraceus* CICC 40330.

Microbial transformation of **72** and three synthetic olean-type pentacyclic triterpenes, 3-oxo oleanolic acid (**93**), 3-acetyl oleanolic acid (**94**) and esculentoside A (**95**) with *Streptomyces griseus* ATCC 13273 and *Aspergillus ochraceus* CICC 40330 was reported by Zhu *et al.* (see Figure 26, Figure 27, Figure 28, Figure 29 and Figure 30). These highly efficient and regioselective methyl oxidation and glycosylation provided an alternative approach to expand the structural diversity of OPTs [38]. The two interesting reactions observed during fermentation of four synthetic pentacyclic triterpenoids (**3**–**4**) with *S. griseus* ATCC 13273 and *A. ochraceus* CICC 40330, are the the regio-selective oxidation of the methyl group on C-4 and C-20 and the formation of glycosyl ester of C-28 carboxyl group. Fermentation of **72** with *S. griseus* ATCC 13273 yielded two more polar metabolites, serratagenic acid (**96**) and 3β,24-dihydroxy-olean-12-en-28,29-dioic acid (**97**). Incubation of **72** with *A. ochraceus* CICC 40330 afforded another polar metabolite, **78** (Figure 26) [38].

Incubation of 3-oxo oleanolic acid (**93**) with *S. griseus* ATCC 13273 produced two polar metabolites, 3-oxo-olean-12-en-28,29-dioic acid (**98**) and 24-hydroxy-3-oxo-olean-12-en-28,29-dioic acid (**99**). On the other hand, incubation of **93** with *A. ochraceus* CICC 40330 afforded a different polar metabolite 28-*O*-β-d-glucopyranosyl 3-oxo-olean-12-en-28-oate (**100**) (Figure 27). The subsequent metabolism of **94** has also been reported. The bacterium *S. griseus* transformed **94** to two polar regioselective products, **96** and **97** (Figure 28). Compound **78**, 28-*O*-β-d-glucopyranosyl,3β-hydroxy-olean-12-en-28-oate was isolated from the culture of *A. ochraceus* CICC 40330 (Figure 28). Another substrate, esculentoside A (**95**) when incubated with *S. griseus* ATCC 13273 for 5 days yieled two less polar products, esculentoside B (**104**) and phytolaccagenin (**105**). Guo *et al.* investigated the regioselective bioconversion of **93** using the fungus *Absidia glauca* and reported the production of three novel hydroxylated metabolites, 1β-hydroxy-3-oxo-olean-11-en-28,13-lactone (**101**), 1*β*,11α-dihydroxy-3-oxo-olean-12-en-28-oic acid (**102**), and 1β,11α,21β-trihydroxy-3-oxo-olean-12-en-28-oic acid (**103**) (Figure 29) [39].

However, the incubation of **95** with *A. ochraceus* CICC 40330 afforded only metabolite **104**. Further metabolism of compound **104** with *S. griseus* ATCC 13273 produced 2β,3β,23,29-tetrahydroxy-olean-12-ene-28,30-dioic acid 30-methyl ester (**106**) and incubation of **105** with *A. ochraceus* CICC 40330 resulted 28-*O*-β-d-glucopyranosyl phytolaccagenin (**107**) (Figure 30) [38].

**Figure 31:**
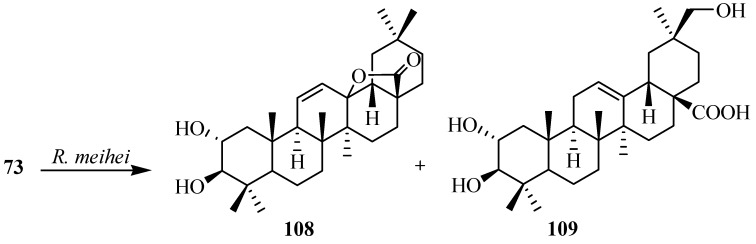
Metabolites isolated from the incubation of maslinic acid (**73**) with *Rhizomucor miehei*.

The pentacyclic triterpene maslinic acid (2α,3β-dihydroxyolean-12-en-28-oic acid, **73**) is a natural pentacyclic triterpenoid compounds which is present in abundant amount in the surface wax on the fruits and leaves of Olea europaea. It is also a byproduct of the solid waste obtained from olive oil production. This compound is also found in *Agastache rugosa*, *Lagerstroemia speciosa*, and *Geum japonicum*. Maslinic acid (**73**) has anti-HIV, anticancer, anti-diabetic, antioxidant and antiatherogenic activities. Martinez *et al.* investigated the metabolism of maslinic acid (**73**) with *Rhizomucor miehei* and reported the production of an olean-11-en-28,13β-olide derivative, 2α,3β-dihydroxyolean-11-en-28,13β-olide (**108**) and a hydroxylated product at C-30 position; 2α,3β,30-trihydroxyolean-12-en-28-oic acid (**109**). These biotransformation reactions are shown in Figure 31 [36].

**Figure 32:**
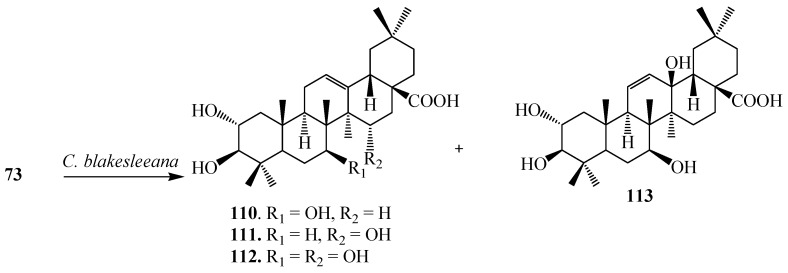
Microbial transformation of maslinic acid (**73**) by *Cunninghamella blakesleeana*.

Feng *et al.* investigated the bioconversion of **73** by *C. blakesleeana* CGMCC 3.910 and demonstrated the formation of four derivatives, 2α,3β,7β-trihydroxyolean-12-en-28-oic acid (**110**), 2α,3β,15α-trihydroxyolean-12-en-28-oic acid (**111**), 2α,3β,7β,15α-tetrahydroxyolean-12-en-28-oic acid (**112**) and 2α,3β,7β,13β-tetrahydroxyolean-11-en-28-oic acid (**113**) (Figure 32) [40].

Ursolic acid (3β-hydroxy-urs-12-en-28-oic acid, UA, **114**), a pentacyclic triterpene acid, exists abundantly in many medicinal plants including sage, rosemary, thyme and lavender [1,41,42]. It displays a remarkable spectrum of biological activities, such as anti-inflammatory activity, anti-allergic activity, antibacterial activity, anti-mutagenicity, hepatoprotective activity, rantimalarial and anti-tumor activity. In addition, UA is also used to induce apoptosis in human liver cancer cell lines, to enhance the cellular immune system and pancreatic β-cell function and to inhibit invasion [1,41].

**Figure 33:**
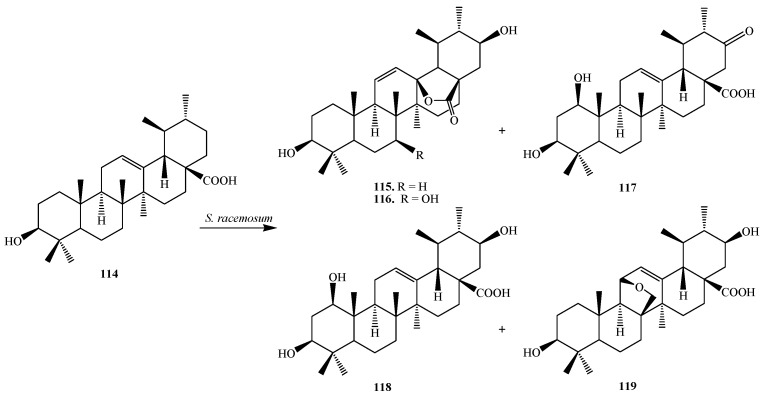
Fermentation of ursolic acid (**114**) with *Syncephalastrum racemosum* (Cohn) Schroter AS 3.264.

Biotransformation of **114** by the filamentous fungus *Syncephalastrum racemosum* (Cohn) Schroter AS 3.264 was reported by Huang *et al.* They reported the regioselective hydroxylation, carbonylation, and condensation reactions. Bioconversion of **114** by *S. racemosum* afforded five metabolites, 3β,21β-dihydroxy-urs-11-en-28-oic acid-13-lactone (**115**), 3β,7β,21β-trihydroxy-urs-11-en-28-oic acid-13-lactone (**116**), 1β,3β-dihydroxy-urs-12-en-21-one-28-oic acid (**117**), 1β,3β,21β-trihydroxy-urs-12-en-28-oic acid (**118**) and 11,26-epoxy-3β,21β-dihydroxyurs-12-en-28-oic acid (**119**) (Figure 33). Compound **117** showed moderate PTP1B inhibitory activity [41].

**Figure 34:**
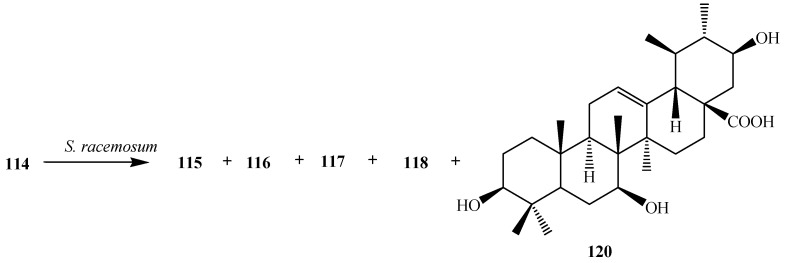
Biotransformation of ursolic acid (**114**) with *Syncephalastrum racemosum* CGMCC 3.2500.

Fu *et al.* conducted microbial transformation of **114** with filamentous fungus *Syncephalastrum racemosum* CGMCC 3.2500 (see Figure 34) and showed the formation of five polar metabolites **115**, **116**, **117**, **118** and 3β,7β,21β-trihydroxy-urs-12-en-28-oic acid (**120**). Metabolites **118** and **120** exhibited anti-HCV activity [42].

**Figure 35:**
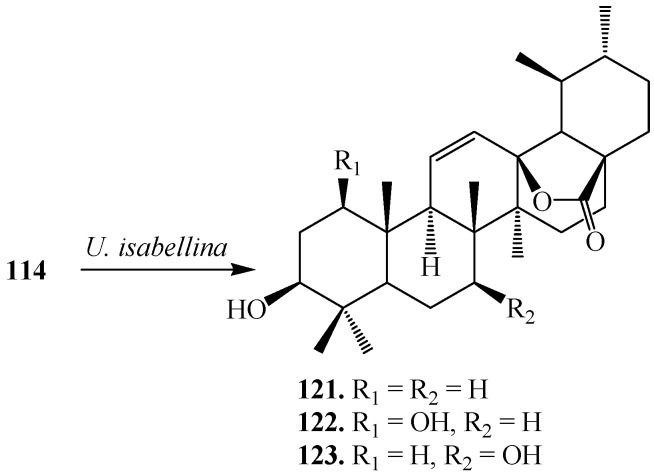
Biotransformation of **114** by an endophytic fungus *Umbelopsis isabellina.*

Endophytic microorganisms are bacterial or fungal organisms which colonize in the healthy plant tissue inter-and/or intracellularly without causing any apparent symptoms of disease. Endophytic microorganisms can produce bioactive natural substances, such as paclitaxel, podophyllotoxin *etc.* [43]*.* Endophytic fungi extensively metabolized 2-hydroxy-1,4-benzoxazin-3(2*H*)-one (HBOA) and biotransformed it to less toxic metabolites probable by their oxidase and reductases. Thus, endophytes attracted more and more attention not only for producing many novel substances but also to biotransform the natural products. Endophytic fungus, *Umbelopsis isabellina*, isolated from medicinal plant *Huperzia serrata*, was utilized to transform **114**, into three regioselective products, 3β-hydroxy-urs-11-en-28,13-lactone (**121**), 1β,3β-dihydroxy-urs-11-en-28,13-lactone (**122**) and 3β,7β -dihydroxy-urs-11-en-28, 13-lactone (**123**) (Figure 35) [44].

**Figure 36:**
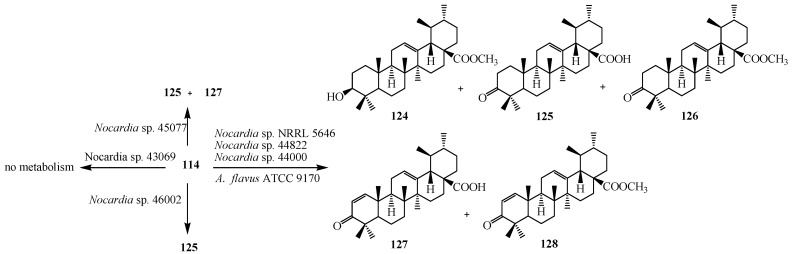
Fermentation of ursolic acid (**114**) with *Nocardia* sp. and *Aspergillus flavus*.

Microbial metabolism of triterpenoid **114** by various *Nocardia* sp. NRRL 5646, *Nocardia* sp. 44822 and *Nocardia* sp. 44000 was investigated by D. Leipold *et al.* Micobial conversion of **114** resulted methyl ester (**124**), ursonic acid (**125**), ursonic acid methyl ester (**126**), 3-oxoursa-1,12-dien-28-oic acid (**127**) and 3-oxoursa-1,12-dien-28-oic acid methyl ester (**128**). *Nocardia* sp. 45077 synthesized ursonic acid (**125**) and 3-oxoursa-1,12-dien-28-oic acid (**127**), while *Nocardia* sp. 46002 produced only ursonic acid (**125**). *Nocardia* sp. 43069 did not cause any biotransformation of this compound [45]. Microbial metabolism of **114** by *Aspergillus flavus* (ATCC 9170) was studied by Ibrahim *et al.*, who showed the formation of 3-oxo ursolic acid derivative, ursonic acid (**125**). These transformation reactions are shown in Figure 36 [46].

**Figure 37:**
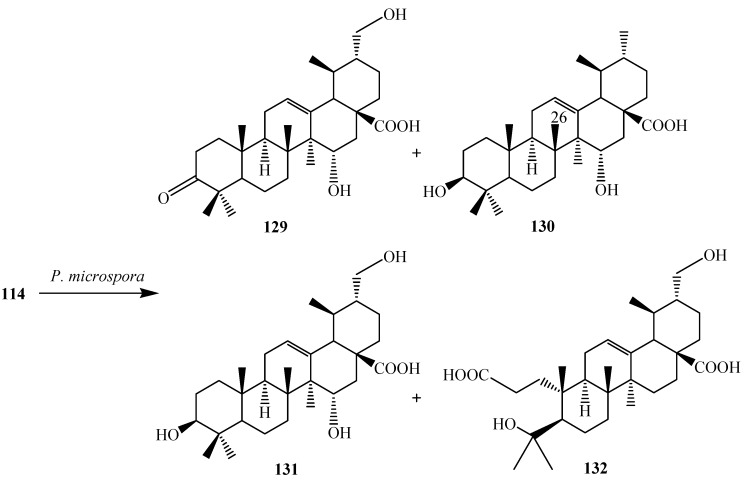
Microbial metabolism of ursolic acid (**114**) with endophytic fungus *Pestalotiopsis microspora*.

Microbial modification of **114** by an endophytic fungus *Pestalotiopsis microspora* was carried out by S. Fu *et al.* Incubation of **114** with *P. microspora* afforded four metabolites: 3-oxo-15α,30-dihydroxy-urs-12-en-28-oic acid (**129**), 3β,15α-dihydroxy-urs-12-en-28-oic acid (**130**), 3β,15α,30-trihydroxy-urs-12-en-28-oic acid (**131**) and 3,4-seco-ursan-4,30-dihydroxy-12-en-3,28-dioic acid (**132**) (Figure 37) [47].

**Figure 38:**
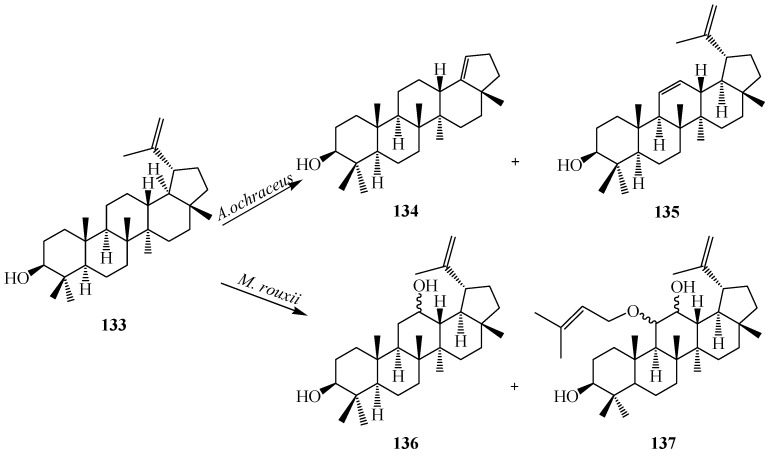
Biotransformation of lupeol (**133**), produced by *Aspergillus ochraceus* and *Mucor rouxii*.

Lupane terpenoids are a group of pentacyclic triterpenoids that consist of compounds with antimalarial, vasorelaxant activities, and potent inhibitors against glycogen phosphorylase. Filamentous fungi, *Aspergillus ochraceus* metabolized lupeol (**133**) to two derivatives **134** and **135** (Figure 38). Fermentation of **133** with *Mucor rouxii* for 240 h yielded two other polar products: **136** and **137** [48].

**Figure 39:**
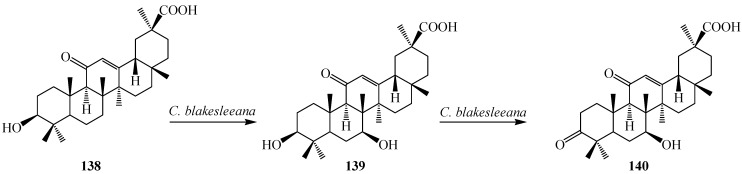
Biotransformation of glycyrrhetinic Acid (**138**) by *Cunninghamella blakesleeana*.

18β-Glycyrrhetinic acid (**138**) is the active form of glycyrrhizin which is the major pentacyclic triterpene found in licorice (*Glycyrrhiza glabra* L.). It is one of the principal constituents of traditional Chinese herbal medicine, the rhizome of *Glycyrrhiza* *uralensis* (called Gancao). Glycyrrhetinic acid (**138**) has been shown to possess several pharmacological activities, such as antiulcerative, anti-inflammatory, immunomodulating, antitumour activities, antiviral activity, interferon-inducibility, antihepatitis effects and anticancer activity. Several hydroxy derivatives of **138** enhanced anti-inflammatory, antioxidant activities, anti-proliferative and apoptotic activities. Several derivatives of **138** have already been used as pharmaceutical drugs [49,50,51]. Many microorganisms catalyze **138** to its derivatives with structural diversity. Qin *et al.* investigated metabolism of **138** with a fungus, *Cunninghamella blakesleeana* AS 3.970 and reported the production of 3-oxo-7β-hydroxyglycyrrhetinic acid (**139**) and 7β-hydroxyglycyrrhetinic acid (**140**) (Figure 39). Both metabolites showed activities against drug-resistant *Enterococcus faecalis* [49]. 

**Figure 40:**
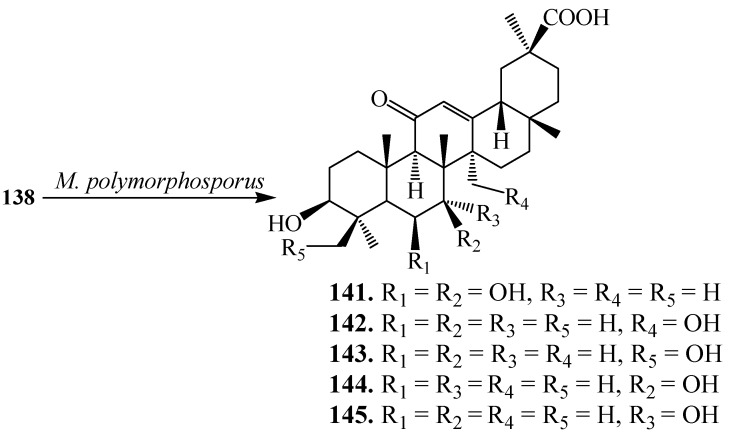
Biotransformation of **138** by *Mucor polymorphosporus*.

Xin *et al.* investigated the microbial transformation of **138** by *Mucor polymorphosporus*. Incubating **138** with *M. polymorphosporus* (Figure 40) for 10 days yielded five polar products: 6β,7β-dihydroxyglycyrrhentic acid (**141**), 27-hydroxyglycyrrhentic acid (**142**), 24-hydroxyglycyrrhentic acid (**143**), 3-oxo-7β-hydroxyglycyrrhentic acid (**144**) and 7α-hydroxyglycyrrhentic acid (**145**) [50].

**Figure 41:**
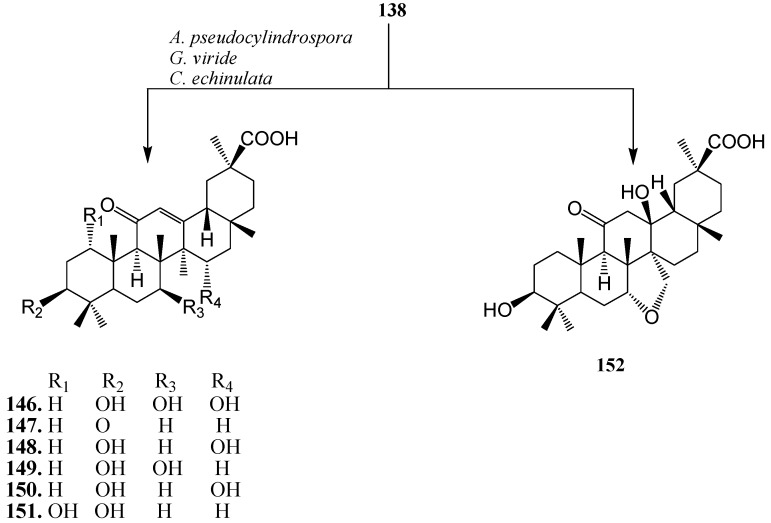
Biotransformation of **138** by *Absidia pseudocylindrospora* ATCC 24169, *Gliocladium viride* ATCC 10097 and *Cunninghamella echinulata* ATCC 8688a.

Bioconversion of **138** using *Absidia pseudocylindrospora* ATCC 24169, *Gliocladium viride* ATCC 10097 and *Cunninghamella echinulata* ATCC 8688a was carried out by G.T. Maatooq *et al.* Fermentation of **138** afforded seven polar derivatives: 7β,15α-dihydroxy-18β-glycyrrhetinic acid (**146**), 3-oxo-18β-glycyrrhetinic acid (**147**), 15-hydroxy-18β-glycyrrhetinic acid (**148**), 7β-hydroxy-18β-glycyrrhetinic acid (**149**), 15α-hydroxy-18α-glycyrrhetinic acid (**150**), 1α-hydroxy-18β-glycyrrhetinic acid (**151**) and 13β-hydroxy-7α,27-oxy-12-dihydro-18β-glycyrrhetinic acid (**152**) (Figure 41). Compound **138**, **146** and **150** enhanced the production of Nitric oxide (NO) in peritoneal rat macrophages treated with CCl_4_ [51].

**Figure 42:**
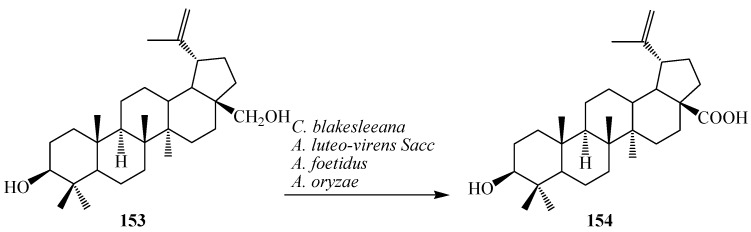
Microbial transformation of betulin (**153**) to betulinic acid (**154**) by Cunninghamella blakesleeana, Armillaria luteo-virens Sacc, Aspergillus foetidus and Aspergillus oryzae.

Betulin (lup-20(29)-ene-3β, 28-diol, **153**), also known as betulinic alcohol, is a pentacyclic triterpene alcohol with a lupane skeleton. The most widely reported source of **153** is the birch tree (*Betula* sp.). It is also called betulinol and is structurally similar to sitosterols. It has estrogenic properties. Betulinic acid (**154**), 3β-hydroxy-lup-20(29)-en-28-oic acid, an antimalarial triterpenoid present in many plant species such as birch tree (*Betula* sp.), *Ziziphus* sp., *Syzygium* sp., *Diospyros* sp. and *Paeonia* sp. [6]. It has attracted more and more attention due to its important physiological and pharmacological activities such as antitumor, anti-HIV, antiviral, anti-leukaemia, anti-inflammatory, antimicrobial, antihelmintic, anti-feedant activities, antimalarial and anticancer activities [6]. Betulinic acid (**154**) and its derivatives are also potential bioactive compounds present in nature [52,53]. Biotransformation of betulin (**153**) was carried out by Chen *et al.* with the filamentous fungi, *Armillaria luteo-virens* Sacc QH (ALVS), *Aspergillus foetidus* ZU-G1 (AF) and *Aspergillus* *oryzae* (AO), which resulted **154** in 6 days. Furthermore, Y. Feng *et al.* investigated biotransformation of **153** to **154** by *Cunninghamella blakesleeana* cells in 6 days of fermentation. These microbial reactions are described rin Figure 42 [52,53].

**Figure 43:**
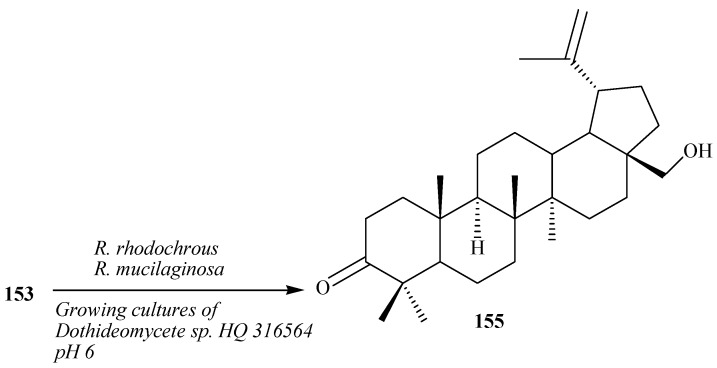
Biotransformation of **153** to betulone (**155**) by *Rhodococcus rhodochrous*, *Rhodotorula mucilaginosa* and growing cultures of marine fungus *Dothideomycete* sp. HQ 316564 at pH 6.

Grishko *et al.* reported regioselective oxidation of **153** into 3-oxo derivative, betulone (**155**) by the resting cells of the actinobacterium *Rhodococcus rhodochrous* IEGM 66 (Figure 43) [54]. Mao *et al.* reported the fermentation of betulin (**153**) by the yeast *Rhodotorula mucilaginosa*. This resulted in the production of compound **155**. Compound **155** exhibited 2 times higher antioxidative activity than that of **153**. Regioselective oxidation of **153** into **155** with growing microorganism cells of marine fungi *Dothideomycete* sp. HQ 316564 was reported by Li *et al.* [55,56].

**Figure 44:**
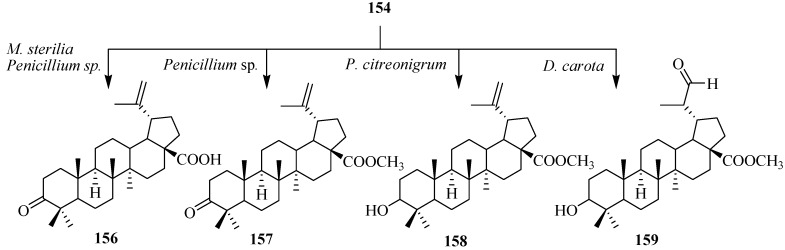
Biotransformation of betulonic acid (**154**) with *Mycelia sterilia*, *Penicillium* sp. *Penicillium citreonigrum* and *Daucus carota* cells suspension.

Preparation of betulinic acid derivatives through groups of fungi, such as *Mycelia sterilia*, *Penicillium citreonigrum* and *Penicillium* sp. was investigated by Baratto *et al.* *M. sterilia* converted **154** to an antimalarial agent [6], betulonic acid (**156**), *Penicillium* sp. biotransformed **154** to **156** and methyl 3-oxolup-20(29)-en-28-oate (**157**), and *P. citreonigrum* transformed **156** to methyl 3-hydroxylup-20(29)-en-28-oate (**158**). Biotranformation of **156** with carrot cell yielded 3-hydroxy-(20*R*)-29-oxolupan-28-oic acid (**159**) in 14 days [57]. These biotransformations are shown in Figure 44.

**Figure 45:**
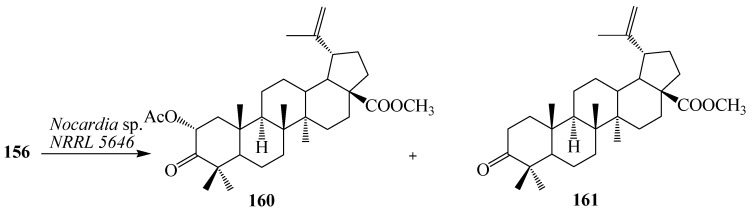
Microbial transformation of **156** by *Nocardia* sp. NRRL 5646.

*Nocardia* sp. NRRL 5646 has been shown to produce a complex set of natural products to generate diverse structures [58]. It has been used extensively to catalyze numerous biotransformations, including carboxylic acid and aldehyde reduction, phenol methylation, and flavone hydroxylation. Microbial transformation of **156** by *Nocardia* sp. NRRL 5646 was investigated by Qian *et al.* Fermentation of **156** for 6 days yielded asymmetric α-hydroxylation product, methyl 2α-acetoxy-3-oxo-lup-20(29)-en-28-oate (**160**) and a methyl esterification of the C-28 carboxyl group, methyl 3-oxo-lup-20(29)-en-28-oate (**161**) (Figure 45) [59].

**Figure 46:**
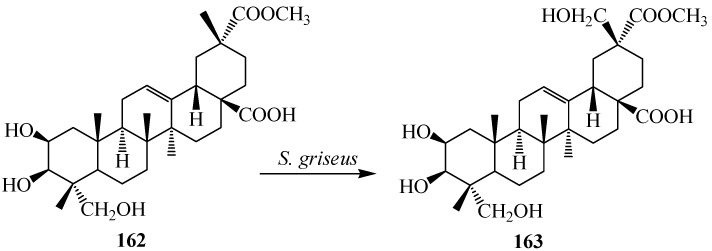
Regio-specific microbial hydroxylation of phytolaccagenin (**162**) by *Streptomyces griseus*.

Phytolaccagenin (2β,3β,23-trihydroxy-olean-12-ene-28,30-dioic acid 30-methyl ester, **162**), a major aglycone constituent found in r*Phytolacca esculenta* van Houtte. *Phytolacca esculenta* is widely distributed in East Asia and is used as a crude drug against edema, theumatism, bronchitis and tumors in China, Korea and Japan. The roots of *P. esculenta* are rich source of saponins and possess anti-inflammatory properties. They also induced immune interferons and tumor necrosis factor [60]. Compound **162** exhibits high activity against acute inflammation. Regiospecific hydroxylation on the C-29 methyl group of **162** by *Streptomyces griseus* ATCC 13273 was reported by Qian *et al.* Fermentation of **162** with *S. griseus* for 96-h afforded one polar metabolite, as 2β,3β,23,29-tetrahydroxy-olean-12-ene-28,30-dioic acid 30-methyl ester (**163**) as shown in Figure 46 [60].

**Figure 47:**
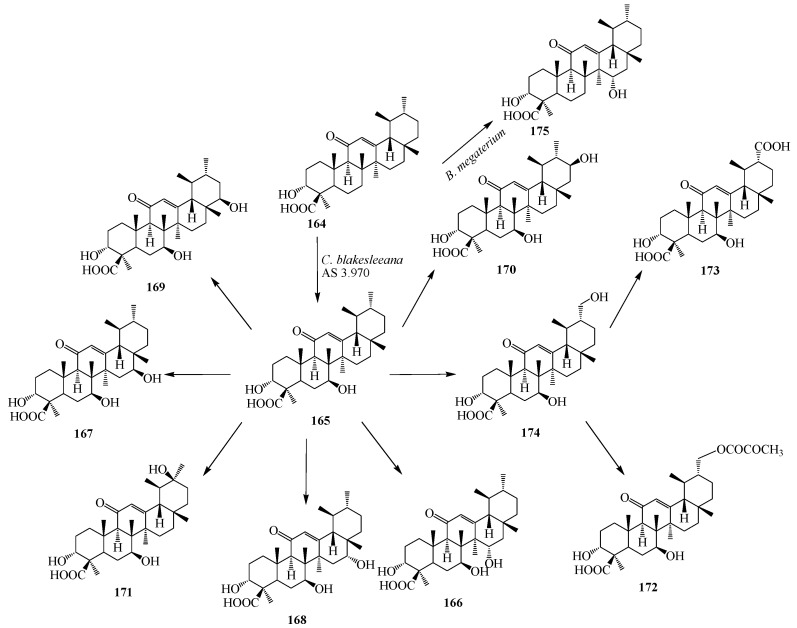
Biotransformation of 11-keto-β-boswellic acid (**164**) by *Cunninghamella blakesleeana* and *Bacillus megaterium*.

The gum resin of *Boswellia serrata* has been used for the treatment of inflammatory and arthritic diseases. Its major active constituents are ursane triterpenoids, boswellic acids (BAs), which include 11-keto-β-boswellic acid (KBA, **164**) (Figure 47), acetyl-11-keto-β-boswellic acid (AKBA), β-boswellic acid (BA) and acetyl-β-boswellic acid (ABA). Among these, AKBA and KBA, possessing an 11-keto group, are the most bioactive compounds. They exhibited significant biological activities, including anti-inflammatory, anti-arthritis, anti-ulcerative colitis, anti-asthma, anticancer, and anti-hepatitis B properties [61]. Microbial rmetabolism of **164** by *Cunninghamella blakesleeana* AS 3.970 was studied by Wang *et al.* Fermentation of **164** by *C. blakesleeana* for 5 days yielded ten regioselective transformed products, which were characterized as 7β-hydroxy-11-keto-β-boswellic acid (**165**); 7β,15α-dihydroxy-11-keto-β-boswellic acid (**166**); 7β,16β-dihydroxy-11-keto-β-boswellic acid (**167**); 7β,16α-dihydroxy-11-keto-β-boswellic acid (**168**); 7β,22β-dihydroxy-11-keto-β-boswellic acid (**169**); 7β,21β-dihydroxy-11-keto-β-boswellicacid (**170**); 7β,20β-dihydroxy-11-keto-β-boswellic acid (**171**); 7β,30-dihydroxy-11-keto-β-boswellic acid (**172**); 3α,7β-dihydroxy-11-oxours-12-ene-24, 30-dioic acid (**173**) and 3α,7β-dihydroxy-30-(2-hydroxypropanoyloxy)-11-oxours-12-en-24-oic (**174**). These fungal transformation reactions are depicted in Figure 47. Compound **167** and **171** exhibit significant inhibitory effect on nitric oxide (NO) production in RAW 264.7 macrophage cells [55]. The location of the hydroxyl functionalities were deduced on the basis of the heteronuclear multiple bond connectivity (HMBC) interactions whereas orientations of OH groups were deduced on the basis of NOESY correlations [61].

Fermentation of **164** with *Bacillus megaterium* based on a recombinant cytochrome P450 system was reported by Bleif *et al.* Metabolism of **164** yielded regio- and stereoselective 15α-hydroxylation of substrate **164** (Figure 47). The structure was identified as 15α-hydroxy-KBA (**175**) by NMR spectroscopy [62].

**Figure 48:**
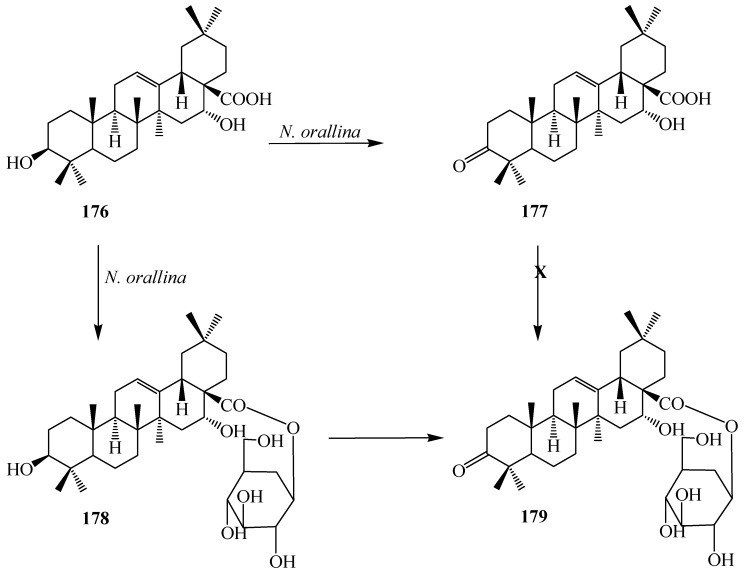
Microbial metabolism of echinocystic acid (**176**) with *Nocardia corallina* CGMCC4.1037.

Hepatitis C virus (HCV) infection is the leading cause of liver fibrosis and cirrhosis which eventually leads to liver cancer. Echinocystic acid (3β,16α-dihydroxy-olean-12-en-28-oic acid, **176**) (Figure 48) is an oleanane-type triterpene, obtained from *Echinocystis fabacea* that exhibits sustantial inhibition of HCV. Echinocystic acid (**176**) and its saponins have been reported to have cytotoxic effects against different cell lines, including the J774.A1, HEK-293, WEHI-164 cell lines, the HepG2, HL-60 cells, the A375, Hela, and L929 cell lines *in vitro*. Echinocystic acid (**176**) and its saponins have many other bioactivities, including anti-HIV activities, antifungal activities, inhibitory activity toward pancreatic lipase, immunostimulatory effect, inhibition of mast cell degranulation, and the interleukin-18 inhibitory activities [63,64].

**Figure 49:**
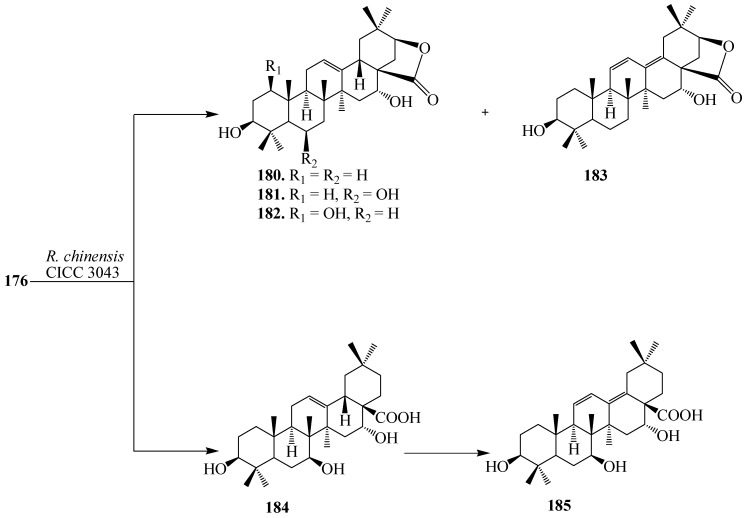
Microbial metabolism of echinocystic acid (**176**) with *Rhizopus chinensis* CICC 3043.

**Figure 50:**
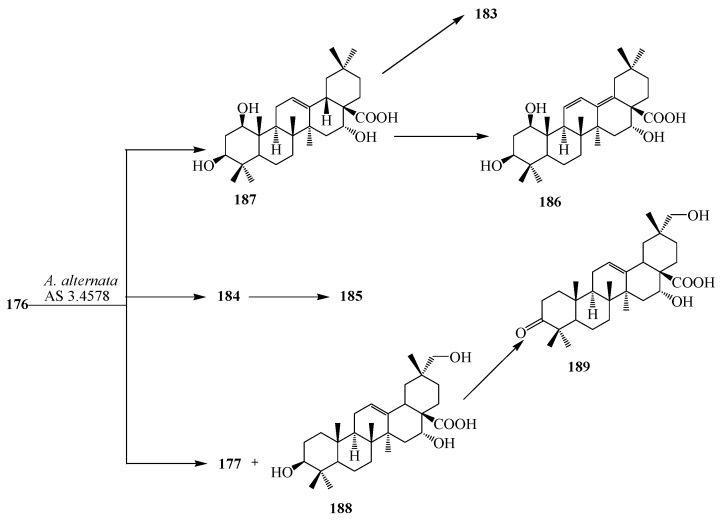
Microbial metabolism of echinocystic acid (**176**) with *Alternaria alternata* AS 3.4578.

Microbial transformation of **176** by *Nocardia corallina* CGMCC4.1037 was reported by Feng *et al.* Incubation of **176** with *N. corallina* CGMCC4.1037 resulted three polar metabolites: 3-oxo-16α-hydroxy-olean-12-en-28-oic acid (**177**), 3β,16α-dihydroxy-olean-12-en-28-oic acid 28-*O*-β-d-glucopyranoside (**178**), and 3-oxo-16α-hydroxy-olean-12-en-28-oic acid 28-*O*-β-d-glucopyranoside (**179**) as described in Figure 48 [63]. Wang *et al.* also reported the regio- and stereoselective modification of **176** by utlizing *Rhizopus chinensis* CICC 3043 and *Alternaria alternata* AS 3.4578 for lead for blocking HCV entry (see Figure 49 and Figure 50) [64]. rThe major product from *R. chinensis* CICC 3043-mediated biotransformation was acacic acid lactone (**180**), along with five minor metabolites: 3β,6β,16α-trihydroxy-olean-12-en-28β-oic acid-21-lactone (**181**), 1β,3β,16α-trihydroxy-olean-12-en-28β-oic acid-21-lactone (**182**), 3β,16α-dihydroxy-olean-11,13(18)-dien-28β-oic acid-21-lactone (**183**), 3β,7β,16α-trihydroxy-olean-12-en-28β-oic acid (**184**) and 3β,7β,16α-trihydroxy-olean-11,13(18)-dien-28β-oic acid (**185**) (Figure 49). Furthermore, *A. alternata* AS 3.4578-mediated metabolism of **176** yielded two major metabolites identified as 1β,3β,16α-trihydroxy-olean-11,13(18)-dien-28β-oic acid (**186**) and **177**, along with five minor metabolites: **183**, **184**, **185**, 1β,3β,16α-trihydroxy-olean-12-en-28β-oic acid (**187)**, 3β,16α,29-trihydroxy-olean-12-en-28β-oic acid (**188)**, and 3-oxo-16α-hydroxy-olean-12-en-28β-oic acid (**189**) as presented in Figure 50 [63,64].

**Figure 51:**
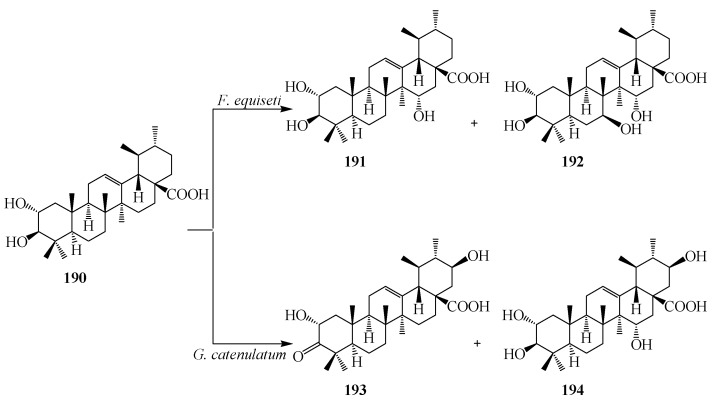
Microbial transformation of corosolic acid (**190**) by *Fusarium equiseti* and *Gliocladium catenulatum*.

Corosolic acid (2β,3α-Dihydroxyurs-12-en-28-oic acid, **190**), a naturally occurring pentacyclic triterpene, has been found in many traditional Chinese medicinal herbs, such as *Lagerstroemia speciosa*, *Eriobotrta japonica*, *Tiarella polyphylla*, *etc.* It has also been found in variety of plants, such as in apples, basil, bilberries, cranberries, and prunes, and has been shown to have a number of biological activities, including suppression of cell proliferation and induction of apoptosis in various cancer cell lines [65,66]. The growing cultures of *Fusarium equiseti* CGMCC 3.3658 and *Gliocladium catenulatum* CGMCC 3.3655 were used for structural modification of corosolic acid (**190**) by Li *et al.* Incubation with *F. equiseti* CGMCC 3.3658 resulted two regioselective hydroxy metabolites 2α,3β,15α-trihydroxyurs-12-en-28-oic acid (**191**) and 2α,3β,7β,15α-tetrahydroxyurs-12-en-28-oic acid (**192**) as shown in Figure 51. *G. catenulatum* CGMCC 3.3655 transformed **190** into 2α,21β-dihydroxy-A-homo-3α-oxours-12-en-28-oic acid (**193**), and 2α,3α,21β-trihydroxyurs-12-en-28-oic acid (**194**) [67] (Figure 51).

## 6. Concluding Remarks and Future Aspects

In summary, microbial transformations are attractive alternative tools for the preparation of bioactive complex triterpenoids, which might be difficult to prepare by conventional chemical routes. They can produce commercially valuable pharmaceuticals for the biorefineries and novel lead molecules towards the development of new drug candidates. The transformation of triterpenoid skeleton through microorganisms in cell cultures exploited regioselective hydroxylations mainly in rings A, B, C, D, E and C-23, C-24, C-29 and C-30 methyl groups, oxidation of C-28 methyl moiety and reduction of C-3 alcohol group, ketones and C=C bond at C-11 and C-12 positions. These modified triterpenoid drugs are currently favored when compared to their natural counterparts due to several therapeutic advantages. Moreover, microbial-catalyzed biotransformations in association with conventional organic synthesis can provide novel routes for the development of new drugs and drug candidates. A number of optimization techniques such as medium, temperature, agitation, pH, *etc.*, have to be established for microbial transformations to be successful and viable. Strain improvement by conventional methods or by genetic engineering identification of alternate biosynthetic routes via microorganisms that have not yet been exploited, new fermentation techniques and optimizing the production facilities will cut the manufacturing cost in future and allow the biotransformation processes to be more competitive to the current synthetic and isolation protocols.
